# Advancing Obstetric Care Through Artificial Intelligence-Enhanced Clinical Decision Support Systems: A Systematic Review

**DOI:** 10.7759/cureus.80514

**Published:** 2025-03-13

**Authors:** Mohammad Omar Abdalrahman Mohammad Ali, Selma Mohammed Abdelgadir Elhabeeb, Nihal Eltayeb Abdalla Elsheikh, Fatima Siddig Abdalla Mohammed, Sulafa Hassan Mahmoud Ali, Aya Abuelgasim Ibrahim Abdelhalim, Dalia Saad Altom

**Affiliations:** 1 Obstetrics and Gynecology, Maternity and Children Hospital, Najran, SAU; 2 Obstetrics and Gynecology, Najran Armed Forces Hospital, Ministry of Defense Health Services, Najran, SAU; 3 Surgical Oncology, Prince Faisal Oncology Center, Buraydah, SAU; 4 General Practice, Najran Armed Forces Hospital, Ministry of Defense Health Services, Najran, SAU; 5 Family Medicine, Najran Armed Forces Hospital, Ministry of Defense Health Services, Najran, SAU

**Keywords:** artificial intelligence, clinical decision support systems, obstetric care, pregnancy care, prenatal care

## Abstract

Although artificial intelligence (AI) has grown over the past 10 years and clinical decision support systems (CDSS) have begun to be used in obstetric care, little is known about how AI functions in obstetric care-specific CDSS. We conducted a systematic review based on research studies that looked at AI-augmented CDSS in obstetric care to identify and synthesize CDSS functionality, AI techniques, clinical implementation, and AI-augmented CDSS in obstetric care. We searched four different databases (Scopus, PubMed, Web of Science, and IEEE Xplore) for relevant studies, and we found 354 studies. The studies were evaluated for eligibility based on predefined inclusion and exclusion criteria. The systematic review incorporated 30 studies after conducting an eligibility assessment of all studies. We used the Newcastle Ottawa Scale for risk bias assessment of all included studies. Medical prediction, therapeutic recommendations, diagnostic support, and knowledge dissemination constitute the key features of CDSS service offerings. The current research on CDSS included findings about early fetal anomaly detection, economical surveillance, prenatal ultrasonography assistance, and ontology development methodologies according to our study findings.

## Introduction and background

Artificial intelligence (AI) has emerged as a transformative force in healthcare, offering innovative solutions to enhance clinical decision-making and optimize patient outcomes [1,2]. In obstetric care, where timely and accurate decisions are crucial for maternal and fetal health, AI-driven clinical decision support systems (AI-CDSS) have gained increasing attention [3]. These systems leverage advanced machine learning algorithms, deep learning networks, and natural language processing (NLP) to assist healthcare professionals in diagnosing conditions, predicting complications, and personalizing treatment strategies [4]. Given the complexity of obstetric management - encompassing prenatal screening, labor monitoring, risk stratification, and postpartum care - AI-enhanced CDSS offers the potential to improve diagnostic accuracy, streamline workflows, and reduce adverse maternal and neonatal outcomes [5].

Obstetric complications such as preeclampsia, gestational diabetes, fetal distress, and preterm birth contribute significantly to global maternal and perinatal morbidity and mortality [6]. Traditional clinical decision support systems (CDSS), while effective in standardizing care, often rely on predefined rules and expert-driven algorithms that may not fully capture the variability and complexity of real-world obstetric scenarios [7]. In contrast, AI-enhanced CDSS dynamically adapts to new data, continuously learning from vast datasets to refine predictions and recommendations [8]. These systems can integrate multimodal data sources, including electronic health records (EHRs), medical imaging, and real-time physiological monitoring, providing clinicians with data-driven insights to support clinical decisions [9].

AI offers promises for obstetric care delivery, but various issues, including untrustworthy functionality and interpretation troubles, discriminatory operation, and ethical dilemmas, persist. AI implementation into clinical practice demands complete validation followed by regulatory certification, while healthcare providers need to show approval before implementation [10]. The unequal distribution of high-quality obstetric AI tools throughout different resource settings creates doubts about fairness in access as well as generalizing benefits [11]. The effectiveness, together with safety aspects and implementation hurdles, of AI-CDSS in obstetrics requires extensive evidence assessment through a unified review of existing research studies.

This systematic review aims to evaluate the impact of AI-enhanced CDSS on obstetric care by synthesizing available evidence on their clinical effectiveness, accuracy, usability, and integration into routine practice. By systematically analyzing studies from diverse sources, this review will identify current trends, benefits, limitations, and future research directions in the adoption of AI-driven decision support in obstetrics. The findings will provide valuable insights for healthcare policymakers, clinicians, and researchers seeking to optimize AI implementation for improved maternal and fetal outcomes.

## Review

Methodology

Review Protocol

This systematic review follows the Preferred Reporting Items for Systematic Reviews and Meta-Analyses (PRISMA) guidelines to ensure methodological rigor and transparency in reporting [12]. The review aims to explore the role of AI-CDSS in advancing obstetric care.

Eligibility Criteria

The inclusion and exclusion criteria were established using the Population, Intervention, Comparison, Outcome, and Study Design (PICOS) framework. The population considered for this review includes pregnant individuals and obstetric healthcare providers who utilize AI-enhanced CDSS. The intervention of interest involves AI-driven CDSS implemented within obstetric care settings. Studies that compare these AI-driven systems to conventional decision-making approaches or traditional CDSS without AI integration were included. The primary outcomes evaluated encompass improvements in maternal and fetal health outcomes, diagnostic accuracy, decision-making efficiency, and patient safety. Eligible study designs include randomized controlled trials (RCTs), cohort studies, case-control studies, cross-sectional studies, and relevant qualitative studies. However, reviews, opinion pieces, and conference abstracts were excluded to maintain the focus on primary research and empirical evidence.

Information Sources

A comprehensive literature search was conducted across four major databases: Scopus, PubMed, Web of Science, and IEEE Xplore. These databases were selected to ensure broad coverage of both medical and technological perspectives on AI-enhanced CDSS in obstetric care. Scopus and Web of Science provided access to a vast range of multidisciplinary research, while PubMed focused on medical literature, ensuring the inclusion of clinically relevant studies. IEEE Xplore was used to identify cutting-edge AI and computational advancements relevant to clinical decision support.

Search Strategy

The search strategy included a combination of Medical Subject Headings (MeSH) terms and keywords related to AI-enhanced CDSS and obstetric care. Boolean operators (AND, OR) were used to refine the search. The primary search terms included "Artificial Intelligence" OR "Machine Learning" OR "Deep Learning," "Clinical Decision Support System" OR "CDSS," and "Obstetric Care" OR "Maternal Health" OR "Perinatal Care." The search was conducted for studies published from 2010 onwards to reflect recent advancements in AI technology. The language was restricted to English. The search strategy was adapted for each database as shown in Table 1.

**Table 1:** Search strategy

Database	Search strategy
Scopus	TITLE-ABS-KEY ("Artificial Intelligence" OR "Machine Learning" OR "Deep Learning") AND TITLE-ABS-KEY ("Clinical Decision Support System" OR "CDSS") AND TITLE-ABS-KEY ("Obstetric Care" OR "Maternal Health" OR "Perinatal Care")
PubMed	("Artificial Intelligence"[MeSH] OR "Machine Learning"[MeSH] OR "Deep Learning") AND ("Clinical Decision Support Systems"[MeSH] OR "CDSS") AND ("Obstetric Care"[MeSH] OR "Maternal Health"[MeSH] OR "Perinatal Care")
Web of Science	TOPIC: ("Artificial Intelligence" OR "Machine Learning" OR "Deep Learning") AND TOPIC: ("Clinical Decision Support System" OR "CDSS") AND TOPIC: ("Obstetric Care" OR "Maternal Health" OR "Perinatal Care")
IEEE Xplore	("Artificial Intelligence" OR "Machine Learning" OR "Deep Learning") AND ("Clinical Decision Support System" OR "CDSS") AND ("Obstetric Care" OR "Maternal Health" OR "Perinatal Care") in Metadata

Study Selection Process

Two independent reviewers manually selected studies based on the predefined eligibility criteria. Titles and abstracts of retrieved records were screened manually, followed by a full-text assessment of potentially eligible studies. To ensure accuracy and consistency, EndNote X9.3.3 (Bld 13966, Clarivate Analytics, Philadelphia, PA, USA) was utilized to identify and remove duplicate records before screening. Any discrepancies in study selection were resolved through discussion or consultation with a third reviewer.

Data Extraction

Data extraction was conducted using a standardized form to ensure consistency and comprehensiveness. The extracted data included study characteristics such as author, year of publication, and country of origin. Additionally, details on the study design, sample size, and population demographics were recorded. Information regarding the AI-CDSS, including the type of AI utilized, its functionality, and its method of integration into obstetric care, was also collected. If a comparative approach was employed, relevant details were noted. The outcomes of interest, encompassing maternal and fetal health indicators, clinical decision-making improvements, and any reported limitations or biases, were systematically documented. This structured approach facilitated a comprehensive synthesis of the evidence gathered.

Risk of Bias Assessment

The risk of bias for included studies was assessed using the Newcastle-Ottawa Scale (NOS). Two reviewers independently performed the quality assessment, and discrepancies were resolved through discussion.

Data Synthesis

Findings were synthesized narratively, categorizing studies based on the type of AI-CDSS, application in obstetric care, and reported clinical outcomes.

Ethical Considerations

Since this study involved secondary data collection from published literature, ethical approval was not required. However, all included studies were assessed for ethical compliance and conflict of interest disclosures.

Results

Search Results

**Figure 1:**
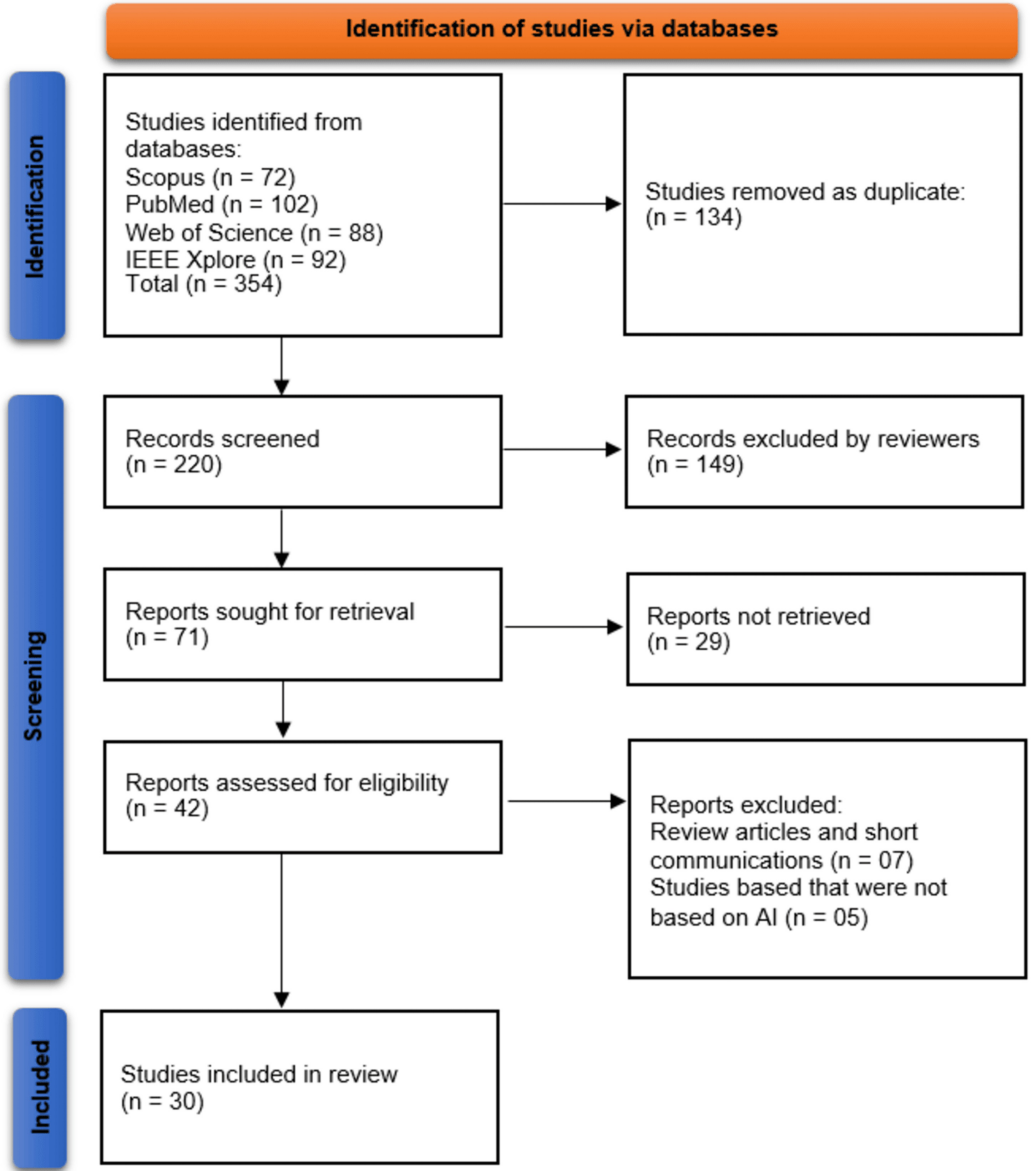
PRISMA flowchart PRISMA: Preferred Reporting Items for Systematic Reviews and Meta-Analyses; AI: artificial intelligence

A total of 354 studies were identified through database searches, including 72 from Scopus, 102 from PubMed, 88 from Web of Science, and 92 from IEEE Xplore. After removing 134 duplicate records using EndNote X9, 220 unique records remained for screening. Following an initial title and abstract review, 149 studies were excluded as they did not meet the predefined eligibility criteria. The full texts of 71 reports were sought for retrieval, of which 29 could not be accessed. The remaining 42 full-text articles were assessed for eligibility, leading to the exclusion of 12 studies - seven review articles or short communications and five studies that did not focus on AI-based CDSS. Ultimately, 30 studies were included in the systematic review for data extraction and synthesis (Figure 1).

Characteristics of Included Studies

The systematic review includes 30 studies that had a range of study designs, including retrospective cohort studies (n = 12), prospective cohort studies (n = 2), predictive modeling studies (n = 3), observational studies (n = 5), and developmental or evaluation studies (n = 8). The sample sizes varied significantly, ranging from 149 to 303,678 participants, reflecting diverse population groups and clinical settings.

The primary objectives of the included studies focused on risk prediction (n = 22), diagnostic support (n = 3), and treatment recommendations (n = 2), with the remainder addressing knowledge representation or software implementation (n = 3). The AI methodologies employed included support vector machine (SVM), random forest (RF), logistic regression, deep learning architectures (convolutional neural network (CNN), long short-term memory (LSTM), gradient boosting (extreme gradient boosting (XGB), light gradient-boosting machine (LightGBM)), and ontology-based models. The majority of studies utilized EHRs (n = 10) or registry data (n = 9), while others relied on surveys, mobile apps, cardiotocograms, and ultrasound databases.

Model validation methods varied, with internal validation performed in 23 studies and external validation in only seven studies, indicating that most studies lacked generalizability beyond their specific datasets. Performance metrics, where reported, highlighted strong classification accuracies, with area under the curve (AUC) values ranging from 0.67 to 0.96, and some models achieving over 90% accuracy in predicting obstetric outcomes.

Despite these advancements, some studies exhibited limitations, such as lack of external validation, small sample sizes, or incomplete data on performance metrics. Nevertheless, the included studies demonstrate the increasing integration of AI-driven decision support tools in obstetric care, highlighting their potential to enhance maternal and fetal health outcomes (Table 2).

**Table 2:** Characteristics of included studies AI: artificial intelligence; ANN: artificial neural network; ACC: accuracy; AUC: area under the curve; BPNN: backpropagation neural network; CDSS: clinical decision support system; CNN: convolutional neural network; CI: confidence interval; EHR: electronic health record; ELM: extreme learning machine; F: F-measure; GBDT: gradient boosted decision tree; GDM: gestational diabetes mellitus; HER: health electronic record; KRAL: knowledge representation and learning; Lasso: least absolute shrinkage and selection operator; LS-SVM: least squares support vector machine; LSTM: long short-term memory; MCC: Matthews correlation coefficient; ML: machine learning; MLP: multilayer perceptron; MRE: mean relative error; NLP: natural language processing; RF: random forest; RBF: radial basis function; ROC: receiver operating characteristic; SVM: support vector machine; XGB: extreme gradient boosting; LightGBM: light gradient-boosting machine

Author	Publishing year	Study design	Objective	Sample size	Data source	Clinical decision support system	AI method	Performance matric	Validation
Hershey et al. [13]	2022	Retrospective cohort study	To estimate the likelihood of spontaneous preterm delivery	2390	Surveys	Risk prediction	SVM	AUC 0.75	Internal
Fernández et al. [14]	2022	Retrospective cohort study	Estimate the delivery method: instrumental vaginal delivery, eutocia vaginal delivery, or cesarean section.	10565	Registry	Risk prediction	MLP, RF, SVM	ACC > 90	Internal
Schmidt et al. [15]	2022	Retrospective cohort study	To forecast unfavorable outcomes for patients who may have preeclampsia.	1647	Registry	Risk prediction	SVM, RF, Ad-aBoost, XBG, and logistic regression	SVM (AUC 0.79)	Internal
Du et al. [16]	2022	Secondary analysis of a cohort study	To predict GDM	565	Registry	Risk prediction	Ad-aBoost, SVM, RF, logistic regression, and XBG	SVM (AUC 0.79)	Internal
Mooney et al. [17]	2021	Retrospective cohort study	To analyze RF to forecast when hypoxic-ischemic encephalopathy will occur.	53000	Registry	Risk prediction	RF	RF (MCC 0.63)	Internal
Tao et al. [18]	2021	Retrospective observational study	To create a hybrid classifier that predicts birth weight.	5759	EHR	Risk prediction	CNN, RF, SVM, LSTM, BPNN, and logistic regression	(MRE 5.65 ± 0.4)	Internal
Escobar et al. [19]	2021	Retrospective cohort study	To estimate the likelihood of maternal, fetal, and neonatal incidents.	303678	EHR	Risk prediction	Gradient boosted, logistic regression	Gradient boosted (AUC 0.786)	External
Tissot and Pedebos [20]	2021	Machine learning-based risk assessment study	To examine embedding techniques for doing risk assessments for miscarriages prior to or during pregnancy.	4676	EHR	Risk prediction	ML, ontology embedding	KRAL (F 0.76)	Internal
Venkatesh et al. [21]	2020	Predictive modeling study	To estimate the chance of postpartum hemorrhage at the time of labor admission.	228438	EHR	Risk prediction	Logistic regression, XGB, F, and Lasso regression	XGB (C statistic0.93; 95% CI 0.92-0.93)	External
Silva et al. [22]	2020	Software architecture design and implementation study	To create a web CDSS for antenatal care guidelines with a minimal and readable syntax.	Not reported	Not reported	Knowledge representation	Ontology	Not reported	Not reported
Ye et al. [23]	2020	Retrospective observational study	To forecast GDM and evaluate their results against logistic regressions.	22242	HER	Risk prediction	Ad-aBoost, LightGBM, logistic regression, voting, XGB, decision tree, RF, gradient boosting, decision tree, and logistic regression	GBDT (AUC 0.74, 95% CI 0.71-0.76)	Internal
Liu et al. [24]	2019	Retrospective cohort study	To predict pregnancy	65276	Mobile app	Diagnostic support	LSTM, Logistic regression	AUC 0.67	External
Wang et al. [25]	2019	Retrospective cohort study	To create a prediction model for postpartum depression with EHR.	179980	HER	Risk prediction	Bayes, RF and XGB	SVM (AUC 0.79)	Internal
Fernandez et al. [26]	2019	Development and evaluation study	To create a decision support system that offers recommendations for ectopic pregnancy treatment at an early stage.	406	HER	Treatment recommendation	Decision tree, SVM, Naïve, and logistic regression	SVM (ACC 0.96)	Internal
Seitinger et al. [27]	2018	Development and validation study	Medical knowledge representation and language processing in obstetrics using Arden syntax.	Not reported	Not reported	Knowledge representation	SVM, Naïve Bayes, multilayer perception, and decision rule	Not reported	Not reported
Fergus et al. [28]	2018	Retrospective observational study	To put vaginal delivery and cesarean sections into different categories.	552	Registry	Risk prediction	Arden syntax	Ensemble (AUC 0.96)	Internal
Maurice et al. [29]	2017	Retrospective observational study	To create a new body of knowledge clever system for imaging with ultrasound.	Not reported	PubMed	Knowledge representation	Ensemble: deferred acceptance, ANN, decision tree, SVM, RF	F 0.71	Internal
Dhombres et al. [30]	2017	Observational study	To create a database with information about ectopic pregnancy.	4260	Ultrasound	Knowledge representation	NLP and Ontology	Precision 0.83	Internal
Paydar et al. [31]	2017	Retrospective analysis	To anticipate the pregnancy results of pregnant women with systemic lupus erythematosus.	149	HER	Risk prediction	NLP and Ontology	MLP (ACC 0.91)	Internal
Ravindran et al. [32]	2015	Observational predictive modeling study	To assess well-being of fetus	2126	Cardiotocogram	Risk prediction	MLP and RBF	ACC 93.61%	External
Jiménez-Serrano et al. [33]	2015	Observational predictive modeling study	To identify postpartum depression in the first week following delivery in the direction of a mobile health app.	1880	Registry	Risk prediction	Ensemble: Bayesian network, SVM, k-NN, and ELM	ANN (ACC 0.79)	Internal
Spilka et al. [34]	2014	Observational cross-sectional study	To analyze the car-diotocogram and assist in making decisions.	634	Cardiotocogram	Diagnostic support	Logistic regression, ANN, SVM, and naïve bayes	Not reported	Internal
Yılmaz and Kılıkçıer [35]	2013	Developmental study	To use the cardiotocogram data to ascertain the fetal condition.	2126	Cardiotocogram	Risk prediction	Latent class analysis	ACC 91.62%	Internal
Ocak [36]	2013	Developmental study	To assess well-being of fetus	1831	Cardiotocogram	Risk prediction	LS-SVM	ACC 99.3%	Internal
Gorthi et al. [37]	2009	Developmental study	To use trends from clinical parameters to forecast pregnancy risk.	200	Synthetic cases	Risk prediction	SVM	ACC 82.5%	Internal
Mueller et al. [38]	2006	Retrospective observational study	To find predictors to help you make the best extubation choices for premature infants.	183	HER	Risk prediction	Decision tree	AUC > 0.9	Internal
Catley et al. [39]	2006	Retrospective observational study	To estimate obstetrical outcomes in populations of low-risk mothers.	48000	Registry	Risk prediction	multiple layer regression and ANN	ROC 0.73	Internal
Goodwin et al. [40]	2000	Retrospective observational study	To predict the preterm birth	19970	HER	Risk prediction	ANN	Customized (AUC 0.75)	Internal
Mongelli et al. [41]	1997	Developmental study with a retrospective observational component	To create an expert system to interpret the acid-base state of the fetal scalp.	2174	Scalp blood Samples	Risk prediction	Neural networks, rule induction, and logistic regression	Not reported	Internal
Woolery and Grzymala-Busse [42]	1994	Retrospective observational study	An expert method for evaluating the risk of premature birth.	18890	Registry	Risk prediction	Decision trees, back-propagation networks, and logistic transformations	ACC 53%-88%	External

Risk of Bias Assessment

The risk of bias for the included studies was assessed using the NOS, which evaluates three key domains: selection (0-4 points), comparability (0-2 points), and outcome (0-3 points). A total score of 9 points indicates the highest quality study with the lowest risk of bias. Overall, the assessment revealed that 13 studies had a low risk of bias (≥8 points), 10 studies exhibited a moderate risk of bias (6-7 points), and 7 studies were classified as high risk of bias (≤5 points). The primary concerns in studies with moderate to high risk were related to retrospective study designs, lack of external validation, and insufficient outcome assessment methods. Despite these limitations, most studies demonstrated methodological rigor, ensuring a reasonable level of reliability in their findings (Table 3).

**Table 3:** Risk of bias assessment using Newcastle-Ottawa scale (NOS) Selection (0-4 points): Evaluates study design, representativeness, and selection criteria. Comparability (0-2 points): Assesses control for confounding factors and study comparability. Outcome (0-3 points): Measures how well outcomes are assessed and reported. Total score (0-9 points): A score of 8-9 indicates low risk, 6-7 suggests moderate risk, and ≤5 reflects high risk of bias.

Study	Selection (0-4)	Comparability (0-2)	Outcome (0-3)	Total score (0-9)	Risk of bias
Hershey et al. [13]	4	2	3	9	Low
Fernández et al. [14]	4	2	3	9	Low
Schmidt et al. [15]	3	2	2	7	Moderate
Du et al. [16]	4	2	3	9	Low
Mooney et al. [17]	3	1	2	6	Moderate
Tao et al. [18]	3	1	2	6	Moderate
Escobar et al. [19]	4	2	3	9	Low
Tissot and Pedebos [20]	3	2	3	8	Low
Venkatesh et al. [21]	4	2	3	9	Low
Silva et al. [22]	2	1	2	5	High
Ye et al. [23]	3	2	3	8	Low
Liu et al. [24]	3	2	2	7	Moderate
Wang et al. [25]	4	2	3	9	Low
Fernandez et al. [26]	3	2	2	7	Moderate
Seitinger et al. [27]	2	1	2	5	High
Fergus et al. [28]	3	2	3	8	Low
Maurice et al. [29]	2	1	2	5	High
Dhombres et al. [30]	3	2	2	7	Moderate
Paydar et al. [31]	3	2	3	8	Low
Ravindran et al. [32]	4	2	3	9	Low
Jiménez-Serrano et al. [33]	3	2	2	7	Moderate
Spilka et al. [34]	3	1	2	6	Moderate
Yılmaz and Kılıkçıer [35]	3	2	3	8	Low
Ocak [36]	3	2	3	8	Low
Gorthi et al. [37]	2	1	2	5	High
Mueller et al. [38]	3	2	2	7	Moderate
Catley et al. [39]	3	2	3	8	Low
Goodwin et al. [40]	2	1	2	5	High
Mongelli et al. [41]	3	1	2	6	Moderate
Woolery and Grzymala-Busse [42]	2	1	2	5	High

Discussion

Research outcomes show that AI-enhanced CDSS has become increasingly important for better maternal and fetal health results. AI prediction models, including machine learning algorithms, SVM, RF, and logistic regression, together with deep learning models (such as CNN and LSTM), have become instrumental tools for obstetricians to forecast maternal health complications and make clinical diagnoses and therapy choices. Most studies examined risk prediction tasks according to the review, and these studies produced high accuracy rates for detecting preeclampsia together with gestational diabetes mellitus (GDM), postpartum hemorrhage, and preterm birth situations.

EHR functioned as the main data source for AI-CDSS in reviewed research because healthcare practitioners are transitioning to decision support using comprehensive patient healthcare data in real-time [21,27]. The application of registry-based studies utilized extensive databases to strengthen model consistency [24]. Most studies demonstrated a detailed weakness because they did not conduct external validation testing which raises questions about whether these models can work reliably with patient groups different from their original development setting [13].

AI Applications in Prenatal and Obstetric Care

Early prevention and intervention are especially crucial when maternal and infant risk factors and anomalies are identified during prenatal care [14,39,42]. Using information from medical records and prenatal care visits, CDSS studies predict the risk of miscarriage, GDM, and unfavorable outcomes from preeclampsia [19,20]. CDSS has also been used in the treatment of ectopic pregnancy, a dangerous condition that frequently results in maternal morbidity and death. Selecting appropriate treatment after diagnosis is a crucial clinical decision-making procedure to prevent additional issues [26]. Following an ectopic pregnancy, machine learning-based CDSS has been explored to help patients and healthcare professionals make more educated clinical decisions [43,44].

**Figure 2:**
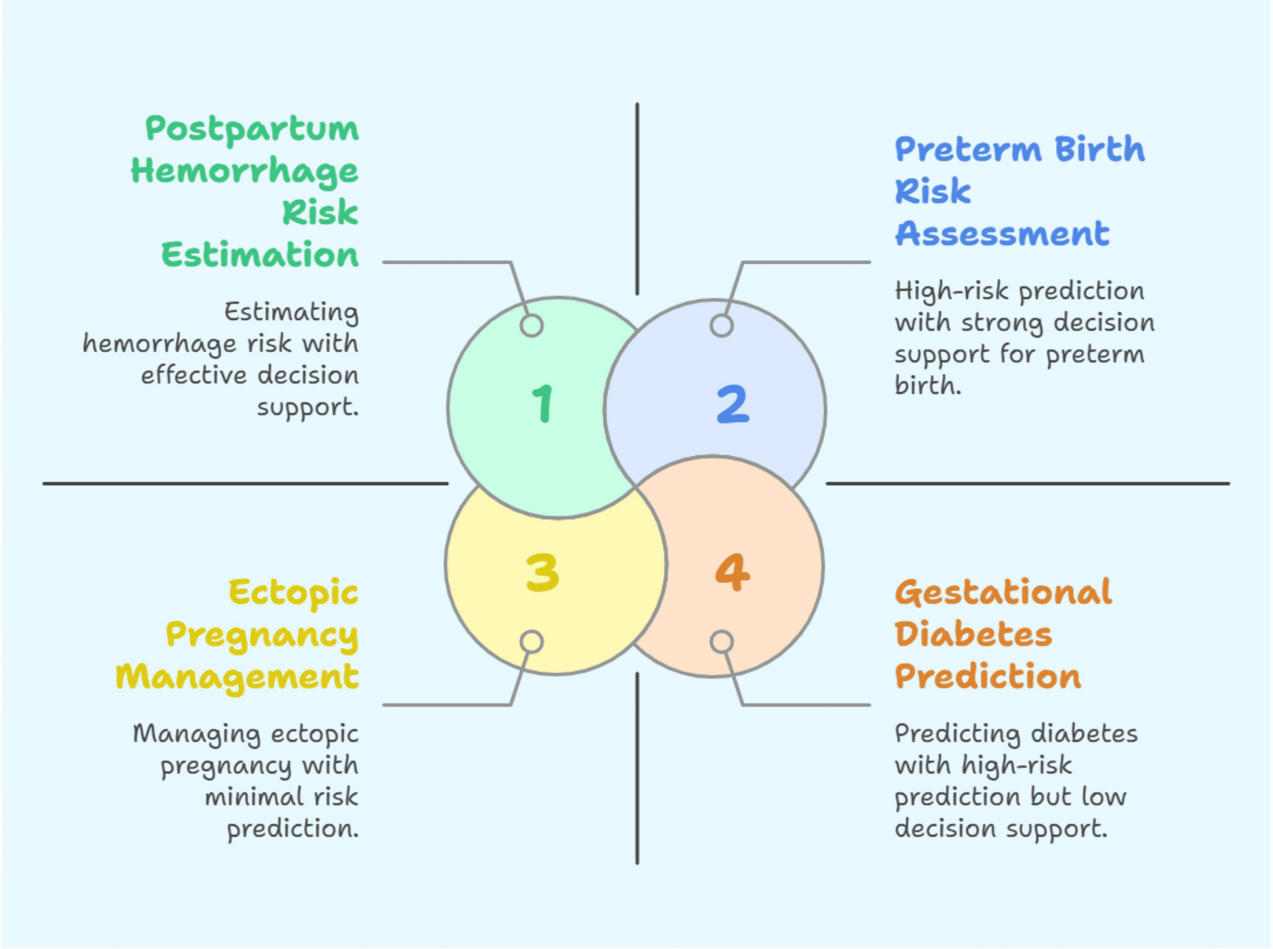
AI-enhanced CDSS application in prenatal and obstetric case Credit: Nihal Eltayeb Abdalla Elsheikh. Created using BioRender (https://app.biorender.com/) AI: artificial intelligence; CDSS: clinical decision support systems

Since the number of pregnant women who suffer from morbidity and mortality is still high and continues to rise in the US and many other nations, more and more CDSS studies are concentrating on creating predictive algorithms for early adverse event detection along with at-risk individuals so that prompt prevention and intervention can be implemented [28]. Prenatal and perinatal advanced healthcare planning can be informed by the identification of persons at risk for preterm birth [33]. Typically, this research included evaluations of risk factors that contribute to adverse occurrences [15]. Machine learning-based studies use feature rating techniques to identify data that are highly predictive of unfavorable outcomes. Interpreting CTG traces to aid in decision-making prior to or during labor and delivery is another example (Figure 2) [31,32].

At the time of labor and delivery admission, CDSS has been used to estimate the risk of postpartum hemorrhage [16,17,29]. A significant contributor to maternal morbidity and mortality, postpartum hemorrhage is responsible for around one-third of birthing people's deaths. Clinical risk estimation has historically relied on the application of parametric statistical models to stratify risk indicators included in patients' medical records [30,36]. To better understand individual diversity and lessen potential biases from conventional guidelines and theoretical frameworks, researchers have tried to include subtleties beyond established risk factors in current CDSS studies. Risk evaluation and screening for postpartum depression, a common but frequently underdiagnosed postpartum illness, is another example [39,40].

AI Methodologies and Applications

While unsupervised algorithms (like clustering) identify decision boundaries lacking a gold standard, supervised algorithms, like classification, prediction, and association rules learning, demand human-annotated evidence as a gold-standard sample, while knowledge-base-independent CDSS usually rely on computational algorithms to learn about decision boundaries [45]. The standard algorithms for clinical forecasting tools, diagnostic support, and medication prescription in this review were largely regression-based algorithms. As benchmarks, some of the research employed parametric linear statistical models [32,35].

SVMs, RFs, and gradient boosting techniques (like XGB) are among the supervised machine learning methods that have gained popularity due to their exceptional performance [26,30]. Simple neural networks, such as artificial neural networks and multilayer perceptrons, have also been tested, particularly when the model's feature space isn't unduly complex and huge in dimensionality [18]. The embedding of ontology was also utilized in the field of pregnancy care to integrate human-curated clinical guidelines and domain-specific medical knowledge into machine-learning models [20]. Furthermore, the discipline has seen the use of deep learning methods (such as recurrent and CNNs), which have produced trained models that outperform other approaches [29,31].

Depending on the knowledge base rules (like fuzzy logic and if-then) or semantic relations (like ontology-defined semantic attributes), they are commonly used in CDSS. For instance, medical picture annotation was accomplished by combining the ontology-based knowledge base with NLP activities, such as named entity recognition and semantic reasoning [38]. Applications of rule-based algorithms have also been shown to be feasible and have strong clinical interpretability [46]. Concerning the creation of knowledge bases in this CDSS research, ontology was frequently employed to build knowledge bases [37].

**Figure 3:**
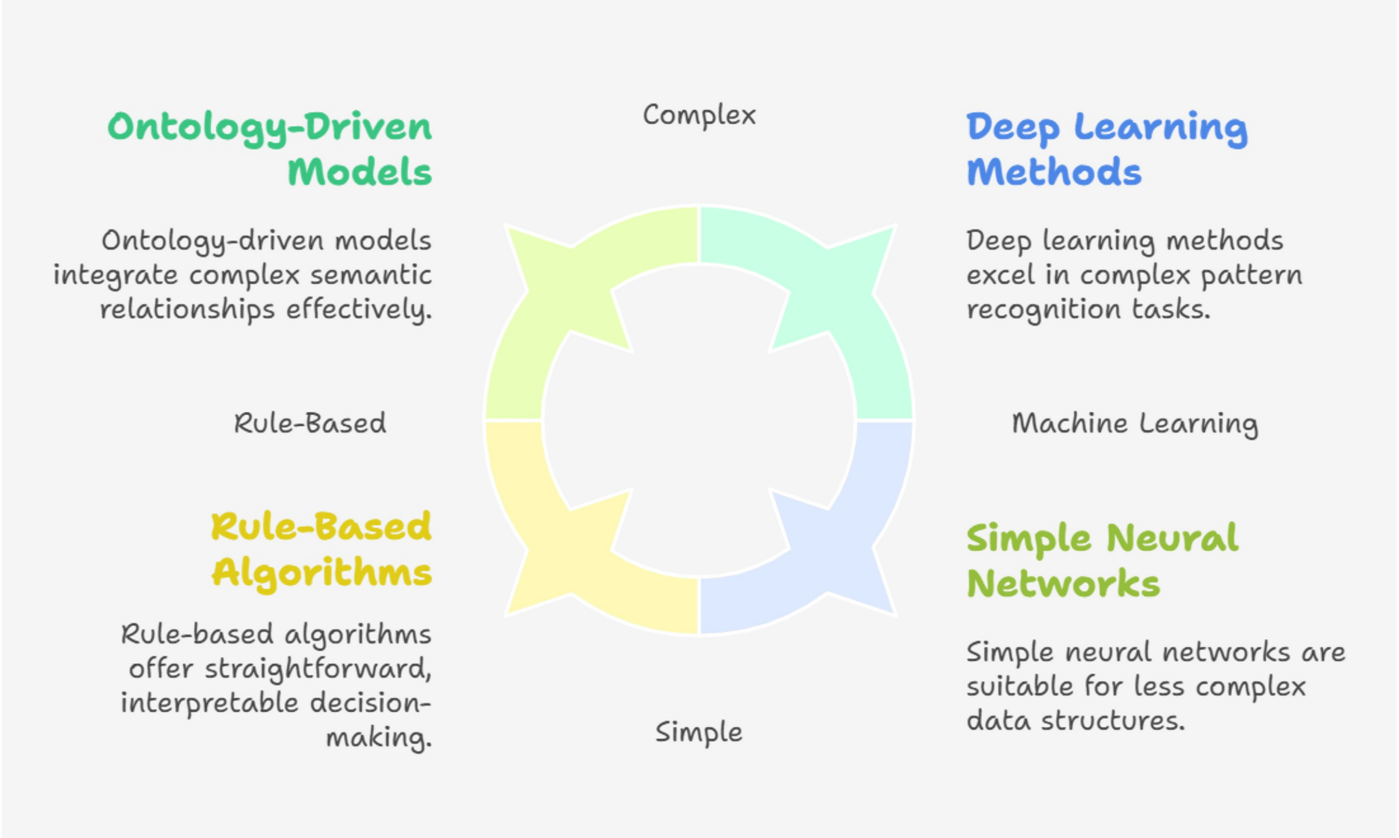
Visual representation of the use of different AI models for clinical decision support systems Credit: Nihal Eltayeb Abdalla Elsheikh. Created using BioRender (https://app.biorender.com/)

To some extent, internal validation has been tested in most of the investigations. Among the validation frameworks employed in the examined research were bootstrap or cross-validation, n-fold cross-validation, and hold-out [39]. Metrics based on probabilistic statistics, such as mean squared prediction error, chi-square, Matthew's correlation coefficient, and c-index; metrics based on descriptive statistics, such as accuracy; and metrics based on statistical theory, such as precision, recall, F measure, and area under the receiver operating characteristics curve, are among the evaluation metrics utilized. The interpretive power of validation was improved in a few studies by using a validation strategy that permitted the computation of confidence intervals [47]. In five of the investigations, external validation was used. These studies typically used distinct data sets, such as those from various clinical sites, to assess CDSS (Figure 3).

Deviated performance and practical clinical judgments arising from CDSS may be caused by biases in data collection, data processing (such as handling missing data and data standardization), ML model development, testing, and algorithm design [32]. Since clinical judgments are based on untrained data, it is argued that poorly sampled data used for training and validation skew AI-augmented systems, of which CDSS may be an example. However, this problem was not addressed in previous research. Appropriate imputation techniques may exclude or magnify biases in samples if biases are not acknowledged or understood. After reviewing, we were unable to locate a thorough discussion or treatment of potential biases in the CDSS design and development process [23].

CDSS Implementation

The last step in integrating research findings into standard practice is CDSS implementation. The conceptual concepts or experimental research designs of clinical execution of stated CDSS have only been covered in a small number of studies [19,27,30]. Web-based data entry and visual result display for CDSS deployment were created for clinical implementation studies. One study used an Android system to illustrate the CDSS interface. Among the evaluated studies, we did not find a thorough CDSS adoption study design (such as usability testing).

Limitations

A number of important limitations need recognition in the context of the systematic review's valuable findings. The research depended largely on retrospective data collection, which introduces possible bias and decreases the study's universal application. Research validity suffers because most studies fail to conduct external validation, which leads to doubts about AI applications throughout different population sectors. The assessment of available studies becomes difficult because of varying approaches to study design and AI techniques and outcomes assessment methods. Studies with positive outcomes are more likely to be published, while screening processes may have influenced the study results. The researchers attempted to incorporate extensive research, but database limitations together with language barriers might have prevented the inclusion of pertinent studies.

## Conclusions

AI-enhanced CDSS holds significant promise in revolutionizing obstetric care by enabling early risk prediction, optimizing labor management, and improving maternal and fetal outcomes. This systematic review confirms that AI-CDSS outperforms traditional models in accuracy and predictive power. However, challenges related to data bias, model validation, clinical integration, and ethical considerations must be addressed before widespread adoption can occur. Future research should focus on refining AI models, ensuring regulatory compliance, and fostering clinician-AI collaboration to unlock the full potential of AI-driven decision support in maternal healthcare.
